# Investigation of parasite genetic variation and systemic immune responses in patients presenting with different clinical presentations of cutaneous leishmaniasis caused by *Leishmania aethiopica*

**DOI:** 10.1186/s40249-024-01244-x

**Published:** 2024-10-16

**Authors:** Endalew Yizengaw, Yegnasew Takele, Susanne Franssen, Bizuayehu Gashaw, Mulat Yimer, Emebet Adem, Endalkachew Nibret, Gizachew Yismaw, Edward Cruz Cervera, Kefale Ejigu, Dessalegn Tamiru, Abaineh Munshea, Ingrid Müller, Richard Weller, James A. Cotton, Pascale Kropf

**Affiliations:** 1 Department of Medical Laboratory Science, College of Medicine and Health Sciences, Bahir Dar University, Bahir Dar, Ethiopia; 2 Institute of Biotechnology, Bahir Dar University, Bahir Dar, Ethiopia; 3 Amhara Public Health Institute, Bahir Dar, Ethiopia; 4 Department of Infectious Disease, Imperial College London, London, UK; 5 Wellcome Sanger Institute, Wellcome Genome Campus, Hinxton, UK; 6 Department of Biology, College of Science, Bahir Dar University, Bahir Dar, Ethiopia; 7 University of Greenwich at Medway, Kent, UK; 8 Nefas Mewcha Hospital, Lay Gayint, Ethiopia; 9 Department of Dermatology, University of Edinburgh, Edinburgh, UK; 10 School of Biodiversity, One Health and Veterinary Medicine, College of Medical, Veterinary and Life Sciences, University of Glasgow, Glasgow, UK; 11 Present Address: Department of Comprehensive Cancer Centre, King‘s College London, London, UK

**Keywords:** Cutaneous leishmaniasis, *Leishmania aethiopica*, Genetics, Cytokines, Chemokines, Plasma

## Abstract

**Background:**

Cutaneous leishmaniasis (CL) is a neglected tropical skin disease, caused by the protozoan parasite *Leishmania*. In Ethiopia, CL is mainly caused by *L*e*ishmania aethiopica* and can present in different clinical forms. The aim of this study was to assess whether these different forms are associated with differences in parasite genetic and host systemic immune signatures.

**Methods:**

Here we analysed the whole genome sequence data for 48 clinical parasite isolates and the systemic immune signature from a cohort of CL patients, who were recruited in Nefas Mewcha, Northern Ethiopia, from January 2019 to January 2022.

**Results:**

Our results show that parasites from CL cases with different presentations in a single Ethiopian setting are from the same genetic population based on a permutation test of genome-wide similarity. Furthermore, a logistic regression test for genome wide association did not identify any individual genetic variants significantly associated with disease presentation. We also measured plasma chemokine and cytokine levels of 129 CL patients presenting with different forms of CL. None of the chemokine [eotaxin, eotaxin-3, interleukin (IL)-8, interferon (IFN)-γ-induced protein-10 (IP-10), monocyte chemoattractant protein (MCP)-1, MCP-4, macrophage-derived chemokines (MDC), macrophage inflammatory protein (MIP)-1α, MIP-1β and thymus- and activation-regulated chemokine (TARC)] or cytokine (IFN-γ, IL-1β, interleukin-2, IL-4, IL-6, IL-10, IL-12p70, IL-13, tumor necrosis factor-α) levels measured were significantly different between the different clinical presentations of CL, as measured by Kruskal–Wallis test. We also compared those with healthy nonendemic controls: our results show a chemokine (IP-10, MCP-1, MCP-4, MDC, MIP-1α, MIP-1β and TARC) but not a cytokine immune signature in patients with CL as compared to healthy nonendemic controls, as measured by Mann-Whitney test.

**Conclusions:**

The results of our study did not identify a systemic immune signature or parasite genetic factors associated with different clinical presentation of CL.

**Graphical abstract:**

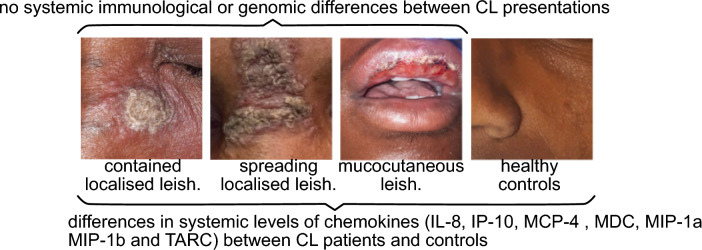

**Supplementary Information:**

The online version contains supplementary material available at 10.1186/s40249-024-01244-x.

## Background

Cutaneous leishmaniasis (CL) is a neglected tropical disease caused by over 20 different species of the protozoan parasite *Leishmania* [1, 2]. It is transmitted to mammalian hosts during the blood meal of infected sandflies. In Ethiopia, CL is mainly caused by *Leishmania aethiopica* [3] with *Phlebotomus longipes* and *P. pedifer* being the most common vectors [4, 5]. The disease presents in three main clinical forms: Localised CL (LCL), where small nodules evolve into ulcerative lesions that usually heal within a few months; mucocutaneous leishmaniasis (MCL), where the lesions affect the nasal or oral mucosa; and diffuse CL (DCL), characterised by numerous non-ulcerating lesions. Both MCL and DCL, as well as persistent LCL rarely heal on their own and require treatment. CL often results in disfiguring scars and can lead to significant social stigma [6].

The mechanisms behind the development of these different presentations of CL are not well understood, and both parasite and host factors could be involved. There has been considerable interest in understanding how variation between parasites might result in differences in the outcome of *Leishmania* infections. The most obvious parasite genetic difference is that only a few *Leishmania* species are associated with visceral disease and a number of factors potentially involved in visceralisation have been identified [7]. However, there are also clear differences between intraspecific parasite isolates and even between clones of a single isolate [8] in both in vitro growth and in infections of animal models [9]. In CL, peroxidase activity has been implicated in the dissemination of MCL-causing *L*. (*V*.) *guyanensis* strain in a rodent model [10]. While most data suggests that parasite isolates from cutaneous and mucosal CL from the same patients are genetically very similar [11, 12], most of this work has relied on experimental characterisation of small numbers of parasite isolates taken from cases with differing clinical presentations.

While whole-genome data from natural populations of many *Leishmania* species is now available [13–16], there has been little work directly attempting to directly relate parasite genetic variation to clinical variation. One exception is a recent genome-wide association study that found some evidence that clinical outcome is linked to parasite genotype in *L. infantum* [16]. Research on *L. aethiopica* is particularly neglected, and the only genomic data available for this species is from 19 historical isolates and some hybrid forms collected from cryopreserved culture collections [17]. For these kinds of samples accurate epidemiological or clinical data is typically not available, and there is often significant variation in sampling times and places which is likely to make detecting clinical associations much more challenging.

In addition to parasite factors, host factors, such as the skin microbiome [18]; vector derived products [19]; the genetics of the host [20]; and immune responses [21] can influence CL development. In contrast to the experimental mouse model of CL [22–24], there are no clearly polarised Th1 and Th2 responses in patients with different clinical manifestations of CL, but a mixed production of cytokines: antigen-specific stimulation of peripheral blood mononuclear cells (PBMCs) from CL patients resulted in production of both interferon (IFN)-γ and interleukin (IL)-4 during the active phase of the disease, but mainly IFN-γ and low IL-4 after healing [25]. There were also increased levels of Th1 type cytokines, such as IFN-γ, IL-2 and tumor necrosis factor (TNF)-α; and Th2 type cytokines, such as IL-4, IL-5 and IL-13; as well as the regulatory cytokine IL-10 in the plasma of *L. guyanensis*-infected CL patients [26]. In patients with mucosal leishmaniasis (ML), the antigen-specific production of IFN-γ and IL-5 was higher as compared to patients with CL; and the detection of IL-4 was low and only present in some ML patients [27]. In a study by Bacellar et al.*,* PBMCs from patients with ML produced increased levels of IFN-γ and TNF-α and decreased levels of IL-10 as compared to patients with CL [28]. It is generally accepted that DCL patients have an inability to mount an efficient immune response [29].

There is very limited information on the immune response in *L. aethiopica*-infected patients or on the mechanisms resulting in the development of different manifestations of CL. It has been shown that *L. aethiopica* parasites isolated from DCL and LCL patients induced different cytokine profiles in PBMCs [30, 31]. A recent study showed that in response to *L. aethiopica*, monocytes and neutrophils from LCL patients were more activated as compared to controls [32], however it was not compared between different clinical forms of CL. Pisa et al.’s study showed that TNF-α was significantly higher in the sera from DCL than LCL patients [33].

The aim of this study was to assess whether different forms of CL were driven by genetic differences between the infecting parasites and whether they were associated with different host systemic immune signatures. To this end we recruited a cohort of CL patients in Nefas Mewcha Hospital, in Lay Gayint District, Northwest Ethiopia [34] and generated whole-genome sequence data from parasites isolated from these patients, as well as measured their plasma cytokine and chemokine profiles. This represents the most detailed immunological and clinical investigation of a large *L. aethiopica* CL cohort to date, and the first attempt to integrate an understanding of the parasite population with clinical and epidemiological data from the same cohort for this species.

## Methods

### CL patients

We have previously described a cohort of CL patients recruited in Nefas Mewcha, Gayint, Northern Ethiopia, from January 2019 to January 2022 [34] and reported that CL patients presented with different clinical forms: localised CL (LCL), mucocutaneous CL (MCL), diffuse CL (DCL), and recidivans (RCL). LCL patients were further divided into two groups: those presenting with a well-defined contained lesion, with a distinct border around the lesion (contained LCL, C LCL) and those presenting with a lesion that did not have clear edges and was spreading (spreading LCL, S LCL). Some CL patients presented with multiple clinical forms of CL (MCL and/or C LCL and or S LCL). For the present study, we analysed whole genome sequences of parasite isolate from 47 CL patients, and plasma cytokines and chemokines from 129 CL patients; all CL patients were from the cohort of patients described in the reference [34]. Patients’ recruitment and diagnosis are detailed in the reference [34]. A further 22 healthy nonendemic controls (HNEC) were also recruited from the staff of Imperial College London, from October 2020 to January 2022.

### Parasite isolates

A skin scraping was obtained from the edge of the lesion with a sterile scalpel and was added to culture medium [M199 medium with 25 mmol/L hepes, 0.2 μmol/L folic acid, 1 mmol/L hemin, 1 mmol/L adenine, 800 μmol/L Biopterin, 50 IU/ml penicillin, 50 mg/ml streptomycin and 10% fetal bovine serum (Merck, Darmstadt, Germany)] and incubated at 26 °C. Parasite growth was examined by microscopy. The promastigotes were then washed with PBS and the DNA was purified using the DNeasy Blood & Tissue Kits (Qiagen, Hilden, Germany) following the manufacturers procedure. The DNA was stored at − 20 °C until further use.

### Genome sequencing and analysis of sequence data

Genomic DNA was isolated from parasite cell pellets using the QIAgen Blood and Tissue DNA kit. The isolated DNA was sheared into 400- to 600-bp fragments by focused ultrasonication (Covaris Inc., Woburn, MA, USA). Sequencing libraries were generated using a PCR-free approach [35] and then cleaned up using Agencourt AMPure XP SPRI beads (Beckman Coulter, Brea, CA, USA). The resulting libraries were sequenced on the Illumina NovaSeq (Illumina Inc, San Diego, CA, USA) platform at the Wellcome Sanger Institute. Low-quality bases at the 3’ end of reads and Illumina sequencing adaptors were removed using Trimmomatic v0.39 [36] with parameters “ILLUMINACLIP:PE_adaptors.fa:2:30:10 TRAILING:15 SLIDINGWINDOW:4:15 MINLEN:50”. Trimmed reads were then mapped against the reference genome of *L. aethiopica* L147 [37] obtained from TriTrypDB release 51 [38] using the mem mapper in the Burrows-Wheeler Aligner (bwa) version 0.7.17 [39] (https://github.com/lh3/bwa). Reads were sorted and duplicates removed using samtools v1.14 (https://github.com/samtools) and picard v 2.22.2 (https://broadinstitute.github.io/picard/). Nucleotide changes and small insertion-deletion variation from the reference were then identified using a pipeline based on the Broad Institute’s Genome Analysis ToolKit (GATK; https://github.com/broadinstitute/gatk/releases), generating per-sample gvcf files with *HaplotypeCaller* assuming diploid genotypes before jointly genotyping across the whole cohort. Single nucleotide polymorphisms (SNPs) were then filtered using hard cut-offs for quality statistics with GATK “VariantFiltration” using the following filters: QD < 2.0; MQ < 50.0; FS > 20.0; SOR > 2.5; BaseQRankSum < − 3.1; ClippingRankSum < − 3.1; MQRankSum < − 3.1; ReadPosRankSum < − 3.1; and DP < 4. Coverage was calculated using samtools v1.17 [40] *depth* command and summaries per sample and per chromosome calculated using GNU datamash v1.2 (https://www.gnu.org/software/datamash/) and R v4.4.1 (https://www.R-project.org/). Samples were retained for further analysis if the median depth of coverage of at least 5 × across the genome and sites were removed if more than 10% of the remaining samples had missing genotype calls for that sites.

Aneuploidy profiles for each sample were estimated by calculating the mean coverage across the genome, excluding chromosome 31, which was assumed to represent the diploid coverage. Per-chromosome mean coverage was divided by this diploid coverage and multiplied by 2 to estimate chromosome-specific coverage, and these estimates confirmed by inspection of the non-reference allele-frequency for each isolate and chromosome, inferred from the per-allele read depth estimates from GATK. Phylogenetic trees were reconstructed using whole-genome nucleotide variation between *L. aethiopica* samples by generating a diploid (heterozygous) consensus genome sequence for each sample by projecting SNP variants onto the reference genome using the *consensus* command in bcftools v1.14 [40] (https://samtools.github.io/bcftools/), phylogenies were inferred using RAxML-NG v0.8.1 [41] (https://github.com/amkozlov/raxml-ng) using the Jukes-Cantor substitution model. Trees were visualized using ggtree v3.8.2 [42] (https://github.com/YuLab-SMU/ggtree/) in R v4.4.3.

Population genomic analysis was based on SNP genotype calls for each sample. Principal-components analysis and tests for differences in genetic similarity within and between phenotype (clinical presentation) groups were performed in PLINK v1.90 [43] (https://www.cog-genomics.org/plink/). Tests for association between individual genetic variants and clinical presentation phenotypes were performed using *–assoc* (for chi-squared test of association) and *–logistic* (for logistic regression tests including PCA axes as explanatory variables) in plink v1.90. In this model, the phenotype for each individual is the dependent variable, and dosage of one allele for each individual is included as an explanatory variable, with scores for each principal component axis as covariates [44]. All other statistical analysis and plotting were performed in R v4.3.1, using tidyverse packages [45] (https://www.tidyverse.org/).

### Blood sample collection

Two ml of venous blood was drawn in heparin tubes and centrifuged, the plasma was collected and immediately frozen to be used at a later time point to measure the levels of cytokines and chemokines.

### Cytokine and chemokine measurements

IFN-γ, IL-1β, IL-2, IL-4, IL-6, IL-8, IL-10, IL-12p70, IL-13, TNF-α, eotaxin, eotaxin-3, interferon-γ-induced protein (IP)-10, monocyte chemoattractant protein (MCP)-1, MCP-4, macrophage-derived chemokines (MDC), macrophage inflammatory protein (MIP)-1α, MIP-1β and thymus- and activation-regulated chemokine (TARC) plasma levels were measured by multiplex assay using Cytokine and Chemokine panel V-PLEX Kits (Meso Scale Diagnostics, Rockville, USA).

### Statistical analysis

A randomisation-based test from PLINK v1.9 (–ibs-test) was used to compare the distribution of similarity scores between isolates for different clinical presentation groups, using the default 100,000 permutations. Tests for association between individual SNPs and clinical presentations used chi-squared and logistic regression approaches implemented in plink, including principal component scores calculated from SNP genotypes in plink. A Shapiro-Wilk test was used to assess the distribution of the data (Table S1). We used non-parametric tests to measure statistical differences and correlations between the different groups as there were always at least one data set that was non-normally distributed. To compare the levels of plasma cytokine and chemokines between the different groups of CL patients, the following tests were used: Mann-Whitney, Kruskal-Wallis and Spearman, using Prism 10 (Graphpad, Boston, USA). Differences were considered statistically significant at P<0.05 and indicated with asterisks as follows: ∗ =*P*<0.05,∗∗ =*P*<0.01,∗∗∗ =*P*<0 .001 and ∗∗∗∗=*P*<0.0001. Unless otherwise stated, summary statistics given are medians followed by interquartile range (IQR) in parentheses.

## Results

### Parasite genetic variation

**Fig. 1:**
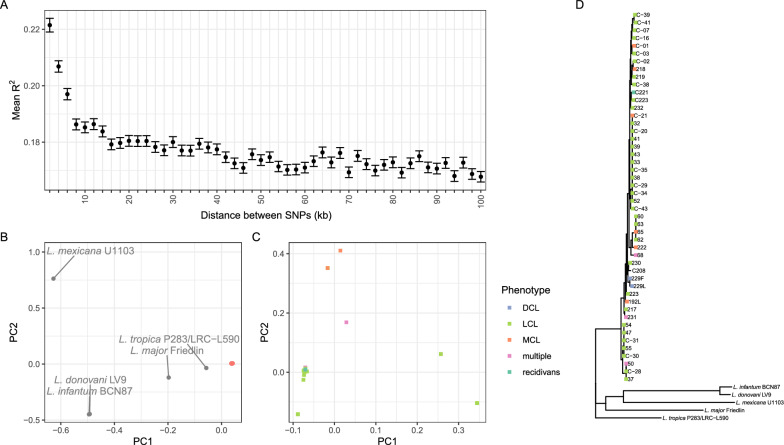
Population genetics of Gayint isolates. **a** Decay of linkage disequilibrium with genomic distance. Symbols show mean R^2^ between pairs of 250,000 random SNPs on all chromosomes, and error bars show 1 standard error for variants in bins of 2 kb distance centred on the indicated distance. R^2^ is calculated for 48 isolates from Gayint district. **b** and **c** Principal components analysis of isolates based on whole-genome SNP data of Gayint isolates **b** with and **c** without outgroups from 5 different *Leishmania* species. **d** Maximum-likelihood phylogeny of CL isolates from Amhara region and comparator isolates based on whole-genome SNP data. Coloured squares on leaves indicate clinical presentation phenotype. Scale bar is in expected number of substitutions per site. *SNP* single nucleotide polymorphism*; PC* principal component; *DCL* diffuse cutaneous leishmaniasis; *MCL* mucocutaneous leishmaniasis; *LCL* localised cutaneous leishmaniasis. The genus name *Leishmania* is abbreviated as *L.* throughout

**Fig. 2:**
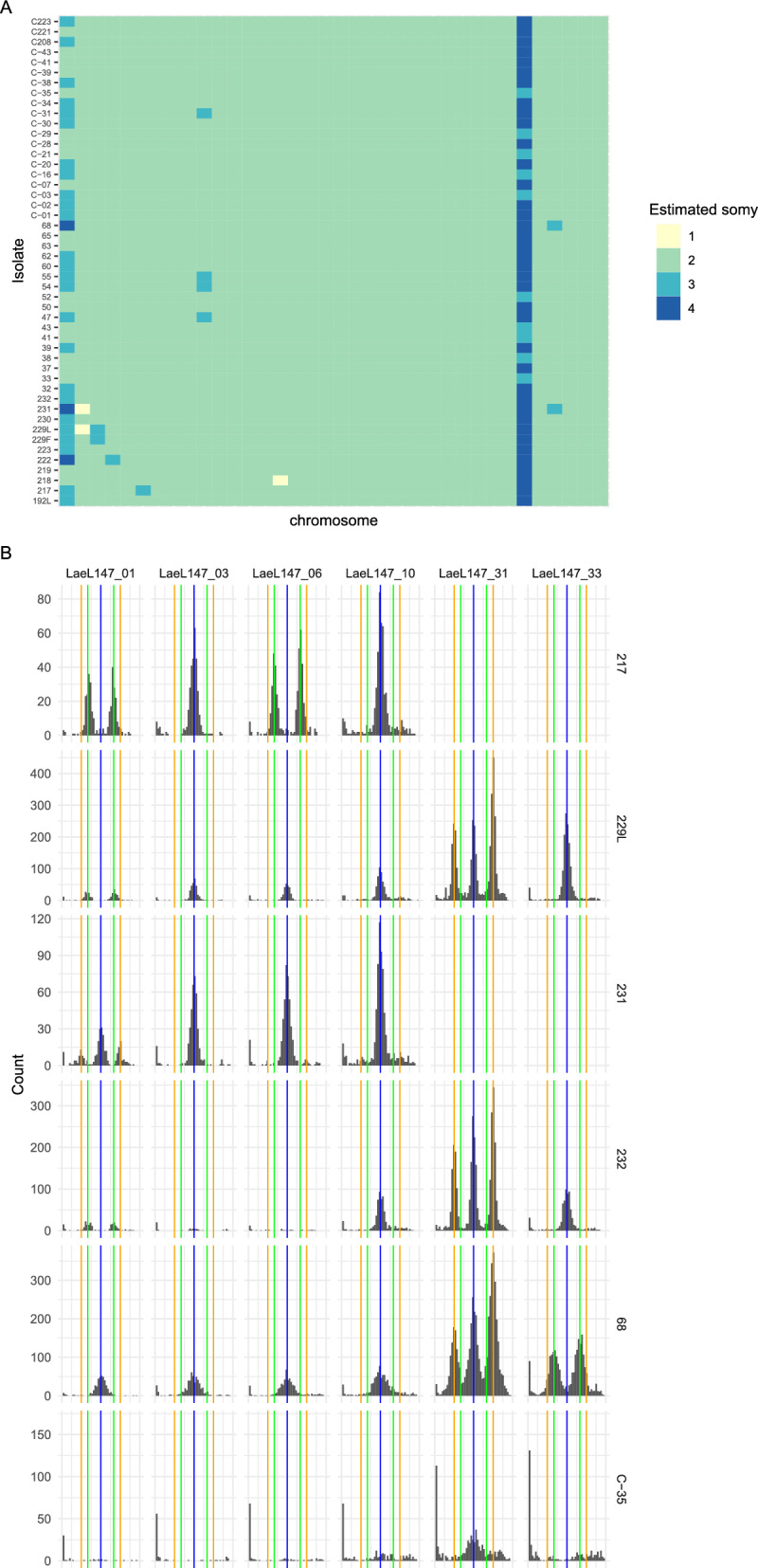
Estimated somy of Gayint isolates and supporting evidence from allele frequency distributions. **a** Estimated chromosome copy number (somy) for all non-outgroup samples based on relative coverage and allele frequency**.** Rows represent individual isolates, columns each chromosome in order from 1 to 36 running left to right. Isolates are ordered by sampling location and clinical presentation. **b** example allele frequency distributions for selected chromosomes and isolates, supporting inferred trisomy and tetrasomies. Each panel is a histogram of non-reference allele frequencies for variants on a particular chromosome for one isolate, with the x-axis showing allele frequencies and the y-axis showing counts of alleles in each allele frequency bin. Blue vertical lines mark 0.5 allele frequency expected from disomy, green lines mark 1/3 and 2/3 allele frequencies expected from trisomy and orange lines mark ¼ and ¾ allele frequencies expected for tetrasomies

We obtained high-quality whole genome sequence data for 48 isolates obtained from the cohort (Table S2). This *L. aethiopica* population showed a moderate level of genetic variation (196,277 variable SNP sites), and phylogenetic and principal components analysis of these data confirm that these parasites are closely related and form a monophyletic group distinct from other *Leishmania* species (Fig. 1b–d). As well as observing limited SNP variation between parasites in this population, we also see remarkably little variation in somy within the *L. aethiopica* population (Fig. 2), with just occasional and sporadic trisomies, particularly for chromosome 1, and a few examples of chromosome loss. Chromosome 31 is observed to be generally tetrasomic, as is general in *Leishmania*. We see decay in linkage equilibrium over tens of kilobases within this set of samples (Fig. 1a) we find a high-to-moderate level of inbreeding in many isolates as frequently seen in *Leishmania* populations (full data not shown). Taken together, these results show that CL in Gayint is caused by a single, interbreeding population of *L. aethiopica*.

### Genetic association with clinical presentation

**Fig. 3:**
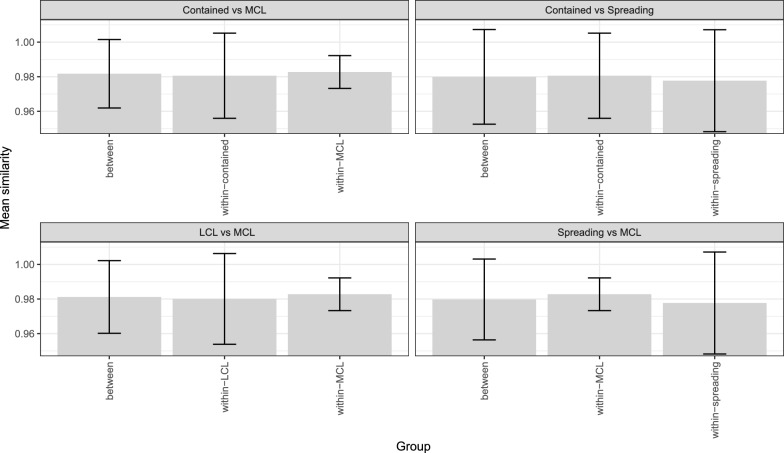
Identity-by-similarity between Gayint isolates within and between groups of isolates with different presentations, based on whole-genome SNP variation data. Shaded bars represent mean similarity of all pairwise comparisons in each category, error bars represent 2 standard deviations. Note that y-axis scale does not start at zero

Of the 48 parasite isolates available, 43 could be classified as coming from patients presenting with either localised (35), mucosal (6) or disseminated (2) CL. The five patients not classified in this way included three with multiple lesion types, one case for which clinical data was not available and a single case of leishmaniasis recidivans. The two parasites isolated from DCL lesions are from lesions on the face and leg of the same patient and genetically very similar (99.97% identical SNP calls at polymorphic sites)–and so were considered likely to be from a single infection and no comparisons with DCL were attempted. There was no significant difference in genome-wide similarity between parasites isolated from patients with similar presentations to those isolated from patients with different presentations (Fig. 3, Table 1). This confirms that the same genotypes of parasites are responsible for causing both disease presentations, rather than particular lineages being associated with specific presentations. For the 35 LCL isolates, we also classified 34 of these into contained (22) and spreading (12) LCL presentation (as described in reference [34]) and again tested for whether parasites isolated from patients with particular forms of LCL were genetically distinct from parasites isolated from patients with different presentations. As before, no difference in overall genetic similarity within and between contained and spreading LCL was identified.

**Table 1:** Comparisons of genome-wide genetic similarity between parasites isolated from patients presenting with different forms of cutaneous leishmaniasis

Group/comparison	Identity-by-similarity	Standard deviation of IBS	*P*-value (test for greater similarity within group than between groups)
LCL	0.980065	0.0131195	0.627744 versus LCL-MCL
			0.50929 versus LCL-DCL
MCL	0.98274	0.00473581	0.545355 versus LCL-MCL
			0.785182 versus MCL-DCL
			0.38874 (S LCL vs. MCL)
			0.553924 (C LCL vs. MCL)
LCL versus MCL	0.98117	0.0104934	N/A
S LCL	0.977682	0.0147224	0.918641 (S LCL vs. C LCL)
			0.689463 (S LCL vs. MCL)
S LCL versus C LCL	0.979914	0.0136714	N/A
S LCL versus MCL	0.979734	0.011677	N/A
C LCL	0.980597	0.0122999	0.312487 (S LCL vs. C LCL)
			0.612314 (C LCL vs. MCL)
			0.502915 (C LCL vs. DCL)
C LCL versus MCL	0.981731	0.00988552	N/A

*LCL* localised cutaneous leishmaniasis; *MCL* mucocutaneous leishmaniasis; *C LCL* contained localised cutaneous leishmaniasis; *S LCL* spreading localised cutaneous leishmaniasis. *N/A* not applicable

Taking advantage of our genome-wide variation data, we also investigated whether individual single nucleotide variants were associated with particular presentations using a genome-wide association study (GWAS) design. For the comparison of LCL with MCL, a single significant SNP was identified at position 676,310 of chromosome 32 (Figure S1a, Table 2). This site is intergenic, lying 73 bp upstream of a putative DNA polymerase subunit (LAEL147_000639500). However, there was evidence that *P*-values from the GWAS were systematically inflated (Figure S1b). This is likely to be due to some remaining population structure, or to linkage between SNP sites. Adjusting for these by including the first 2 principal component of genetic variation as covariates in the analysis removed the *P*-value inflation, but the identified SNP was no longer significant in this analysis (Table 2). We also compared parasites from the two LCL presentation types against MCL and against each other but found no significant associations with SNP variants in these comparisons (Table 2).

**Table 2:** Summary of genome-wide association results

Comparison	Chi-squared test			Logistic regression (2 PCs)		
	Raw *P*-value of most significant variant	Corrected *P*-value of most significant variant	Number of SNPs with corrected *P*-value below 0.01	Raw *P*-value of most significant variant	Corrected *P*-value of most significant variant	Number of SNPs with corrected *P*-value below 0.01
LCL versus MCL	2.79 × 10^–9^	0.00052	1	0.004947	1	0
S LCL versus C LCL	0.00019	1	0	0.006731	1	0
S LCL versus MCL	1.66 × 10^–5^	1	0	0.01816	1	0
C LCL versus MCL	1.66 × 10^–5^	1	0	0.01011	1	0

*LCL* localised cutaneous leishmaniasis; *MCL* mucocutaneous leishmaniasis; *C LCL* contained localised cutaneous leishmaniasis; *S LCL* spreading localised cutaneous leishmaniasis

We also tested for differences in chromosome copy number between parasites of different presentations, using a Fisher’s exact test to test for association between somy at each chromosome where there was variation in copy number and presentation phenotypes for each pair of presentations compared in the SNP-based GWAS. We found no significant associations (Table S3).

### Chemokine and cytokine profile

Chemokines and cytokines were measured in the plasma collected from CL patients, that were part of the cohort described in reference [34], and from healthy nonendemic controls. The cohort of CL patients consisted of 96 LCL and 33 MCL patients. Of the 96 LCL patients, 65 presented with contained lesions (C LCL) and 31 with spreading lesions (S LCL). 41 patients were females and 88 were males. The median age of the female CL patients was 32 (20–49) years, and that of males was 31 (20–45) years (*P* = 0.9636, as measured by Mann-Whitney test). There were no significant differences between the duration of illness, the BMI or parasite grading as described in between C LCL, S LCL and MCL patients (Table 3, as measured by Kruskal-Wallis test). However, S LCL patients presented with significantly more lesions as compared to C LCL and MCL patients (Table 3, as measured by Dunn’s multiple comparisons test) [34].

**Table 3:** Clinical characteristics of cutaneous leishmaniasis patients

	C LCL (*n* = 65)	S LCL (*n* = 31)	MCL (*n* = 33)	*P-value	CL	^*P*-value
Duration of illness (months)	9 (6–12)	12 (7–12)	9 (5–12)	0.2750	N/A	N/A
BMI (kg/m^2^)	21.1 (19.4–22.8)	21.4 (19.1–22.7)	20.0 (19.1–21.7)	0.5146	N/A	N/A
Number of lesions	1 (1–1)	2 (1–4)	1 (1–1)	0.0002	C versus S	0.0003
					C versus MCL	> 0.9999
					S versus MCL	0.0011
Parasite grading ( +)	1 (1–2)	1 (1–2)	1 (1–2)	0.7844	N/A	N/A

Statistical differences in duration of illness, BMI, number of lesions and parasite grading between C LCL, S LCL and MCL were tested by Kruskal–Wallis test (*) and when the *P*-value for the Kruskal–Wallis test was positive, statistical differences between specific CL presentations were tested by Dunn’s multiple comparisons test (^)

*MCL* mucocutaneous leishmaniasis; C = *C LCL* contained localised cutaneous leishmaniasis; S = *S LCL* spreading localised cutaneous leishmaniasis; *N/A* not applicable

#### Chemokines

We first measured the levels of chemokines in the plasma of CL patients presenting with different clinical forms and compared them to those of healthy non endemic controls (HNEC). Whereas plasma levels of Eotaxin (*P* = 0.2269), Eotaxin-3 (*P* = 0.9983) and MCP-1 (*P* = 0.5721) were similar between CL patients and controls, there were significantly higher levels of IL-8 (*P* < 0.0001), IP-10 (*P* < 0.0001), MCP-4 (*P* < 0.0001), MDC (*P* < 0.0001), MIP-1α (*P* = 0.0019) MIP-1β (*P* < 0.0001) and TARC (*P* < 0.0001) in the plasma of CL patients as compared to HNEC (Table 4, as measured by Mann-Whitney test). There were higher levels of MCP-4 in the plasma of patients with MCL and C LCL, but not S LCL and higher levels of MIP-1α in the plasma of C LCL, but not S LCL or MCL patients as compared to HNEC. No significant differences were observed between C LCL, S LCL and MCL patients (Table 4, as measured by Kruskal-Wallis and Dunn’s multiple comparisons tests).

**Table 4:** Comparison of plasma chemokine levels between cutaneous leishmaniasis patients presenting with different clinical forms and healthy nonendemic controls

Chemokines (pg/ml)			*P-value	Dunn’s multiple comparisons	^*P*-value
Eotaxin	MCL	1738 (218–2373)	0.1348	N/A	N/A
	C LCL	1219 (302–1962)			
	S LCL	709 (233–1500)			
	HNEC	1103 (557–1426)			
Eotaxin-3	MCL	65.4 (12.9–121.3)	0.2935	N/A	N/A
	C LCL	42.0 (12.1–96.4)			
	S LCL	27.6 (12.0–64.4)			
	HNEC	46.5 (17.0–90.6)			
IL-8	MCL	11.8 (7.9–20.3)	0.0006	MCL versus C	> 0.9999
	C LCL	12.2 (7.4–19.7)		MCL versus S	> 0.9999
	S LCL	11.2 (8.2–18.6)		MCL versus HNEC	0.0018
	HNEC	7.4 (5.6–8.4)		C versus S	> 0.9999
				C versus HNEC	0.0010
				S versus HNEC	0.0027
IP-10	MCL	544 (446–957)	< 0.0001	MCL versus C	> 0.9999
	C LCL	489 (394–718)		MCL versus S	0.3636
	S LCL	444 (318–624)		MCL versus HNEC	< 0.0001
	HNEC	241 (197–340)		C versus S	0.9757
				C versus HNEC	< 0.0001
				S versus HNEC	0.0030
MCP-1	MCL	183 (124–266)	0.0652	N/A	N/A
	C LCL	157 (119–214)			
	S LCL	131 (105–178)			
	HNEC	178 (130–205)			
MCP-4	MCL	395 (155–529)	0.0004	MCL versus C	> 0.9999
	C LCL	325 (173–539)		MCL versus S	> 0.9999
	S LCL	219 (128–383)		MCL versus HNEC	0.0018
	HNEC	128 (62–188)		C versus S	> 0.9999
				C LCL versus HNEC	0.0003
				S LCL versus HNEC	0.0754
MDC	MCL	674 (599–890)	< 0.0001	MCL versus C	> 0.9999
	C LCL	764 (647–987)		MCL versus S	0.9081
	S LCL	815 (628–1027)		MCL versus HNEC	< 0.0001
	HNEC	390 (347–484)		C versus S	> 0.9999
				C LCL versus HNEC	< 0.0001
				S LCL versus HNEC	< 0.0001
MIP-1α	MCL	15.8 (11.0–20.4)	0.0063	MCL versus C	0.5369
	C LCL	18.6 (12.6–24.9)		MCL versus S	> 0.9999
	S LCL	15.4 (10.5–25.0)		MCL versus HNEC	0.4567
	HNEC	11.8 (9.9–14.6)		C versus S	> 0.9999
				C versus HNEC	0.0038
				S LCL versus HNEC	0.1146
MIP-1β	MCL	102.2 (69.2–150.0)	< 0.0001	MCL versus C	> 0.9999
	C LCL	101.1 (67.3–142.8)		MCL versus S	> 0.9999
	S LCL	122.6 (73.7–189.6)		MCL versus HNEC	< 0.0001
	HNEC	51.5 (42.6–62.5)		C versus S	> 0.9999
				C versus HNEC	< 0.0001
				S versus HNEC	< 0.0001
TARC	MCL	195 (66–647)	< 0.0001	MCL versus C	> 0.9999
	C LCL	289 (142–525)		MCL versus S	0.7679
	S LCL	269 (139–615)		MCL versus HNEC	0.0009
	HNEC	66 (41–87)		C versus S	> 0.9999
				C versus HNEC	< 0.0001
				S versus HNEC	< 0.0001

Chemokine levels were measured in the plasma isolated from the blood of MCL (*n* = 33), C LCL (*n* = 65), S LCL (*n* = 29) and HNEC (*n* = 22) by multiplex assay, as described in Materials and Methods

Statistical differences in chemokines levels (pg/ml) between C LCL, SLCL, MCL and HNEC were tested by Kruskal–Wallis test (*) and when the *P*-values for the Kruskal–Wallis test were positive, statistical differences between specific CL presentations were tested by Dunn’s multiple comparisons test (^)

*MCL* mucocutaneous leishmaniasis; C = *C LCL* contained localised cutaneous leishmaniasis; S = *S LCL* spreading localised cutaneous leishmaniasis; *HNEC* Healthy nonendemic controls; *N/A* not applicable

#### Cytokines

Of all the cytokines tested, only IFN-γ and TNF-α were above the detection limit (Table 5). No significant differences were observed in plasma levels of IFN-γ and TNF-α between the different forms of CL (MCL, C LCL and S LCL) patients and HNEC (Table 5, as measured by Kruskal-Wallis and Dunn’s multiple comparisons tests). Of note, when all the C LCL and S LCL patients were pooled, the levels of TNFα were marginally higher in the plasma of LCL patients as compared to MCL patients (*P* = 0.0359, as measured by Mann-Whitney test, Table S4).

**Table 5:** Comparison of plasma cytokine levels between cutaneous leishmaniasis patients presenting with different clinical forms and healthy nonendemic controls

			*P*-value
IFNγ (pg/ml)	MCL	6.2 (3.8–10.3)	0.2551
	C LCL	7.0 (4.9–13.5)	
	S LCL	6.2 (4.6–12.5)	
	HNEC	5.6 (3.5–9.1)	
TNFα (pg/ml)	MCL	0.7 (0.6–0.9)	0.0903
	C LCL	0.9 (0.7–12)	
	S LCL	0.8 (0.7–1.1)	
	HNEC	0.8 (0.7–0.9)	

Cytokine levels were measured in the plasma isolated from the blood of MCL (*n* = 33), C LCL (*n* = 65), S LCL (*n* = 31) and HNEC (*n* = 22) by multiplex assay, as described in Materials and Methods

Each symbol represents the value for one individual, the straight lines represent the median. Statistical differences were determined using a Kruskal–Wallis test. *MCL* mucocutaneous, *C LCL* contained localised cutaneous leishmaniasis, *S LCL* spreading localised cutaneous leishmaniasis patients, *HNEC* healthy nonendemic controls

### Correlation between plasma chemokine and cytokine levels and clinical parameters

The clinical parameters collected from these patients were wide-ranging: the age of the CL patients varied from 18 to 68 years; the BMI from 16.3 to 27.3 kg/m^2^; the numbers of lesions from 1 to > 5; the parasite gradings from 1 + to 6 + as described in the reference [46]; and the duration of illness from 1 to 180 months. Therefore, we investigated if there was any association between these parameters and the levels of plasma cytokines and chemokines. Correlations, as measured by Spearman test, were observed between age and the levels of IP-10 (*P* = 0.0089, r = 0.3270) and MCP-1 (*P* = 0.0125, r = 0.3127) in C LCL patients; between the number of lesions and Eotaxin (*P* = 0.0456, r = 0.2508); between parasite grading and Eotaxin (*P* = 0.0120, r = -0.5041), Eotaxin-3 (*P* = 0.0040, r = -0.5640) and MCP-1 (*P* = 0.0112, r = -0.5081) in MCL patients (Table S5); and between duration of illness and MIP-1α (*P* = 0.0021, r = -0.3797) in C LCL patients. None of the other correlations were significant (Table S5).

## Discussion

Here, we present the first whole-genome data from recent clinical isolates of *L. aethiopica* sampled in a well-described epidemiological context and accompanied by clinical and immunological data. Across *Leishmania* species, genomic data is only available for a few collections of recent clinical isolates [14, 16, 47, 48]. The only previously available genomic data for *L. aethiopica* apart from the reference genome assembly [37] was from historical cryopreserved isolates [17], although microsatellite and RFLP data from small numbers of isolates has been published [49, 50]. We find limited genetic diversity across the set of isolates, although this is still very much more diverse than the largely clonal population responsible for most VL cases in the Indian subcontinent [48] but less so than in the *L. braziliensis* species complex [14]. We find rapid decay in linkage disequilibrium within these isolates (as found across the species [17]). These isolates thus likely represent a single, interbreeding population of related *L. aethiopica* strains causing cutaneous leishmaniasis in Gayint. We find no evidence that other *Leishmania* species are involved in CL in Gayint as has been reported in Ethiopia [51] and elsewhere in Africa [52]; Kenya [53] and in Yemen [54].

As in previous work [50], we find no significant genetic differentiation between parasites causing different clinical presentations of CL. The availability of genome-wide variation data allowed us to test for association between single nucleotide variants and clinical presentations. In a genome-wide association test with these data, we also did not identify a convincing signature of association between any single nucleotide variation and clinical presentation. The single intergenic SNP significantly associated with MCL in contrast to LCL cases did not remain significant when attempting to correct for population structure within this set of isolates. In any case, the functional relevance of a non-coding SNP variant is unclear, even if this is sufficiently close to the downstream gene (a putative DNA polymerase subunit) to be part of the 5’ UTR and thus have a potential role in mediating gene expression [55]; although the role of these elements is much less well-defined than for 3’ UTRs. While we might expect a more profound parasite difference between the relatively localised infections in LCL and MCL and the more widely disseminating DCL, the low prevalence of DCL in this setting [34] makes it challenging to assemble a sufficiently large patient cohort to identify variation associated with this presentation. It is not clear whether the extensive spreading of the lesions in DCL patients are due to parasites disseminating from one sand fly bite or whereas it might result from different sand fly bites. Our results show that the parasites isolated from two distant sites (face and leg) from one DCL patient were genetically very similar, suggesting that the same parasites were responsible for the extensive spread of lesions in this patient.

Our results identify a chemokine signature in patients with CL as compared to HNEC. The levels of IL-8, IP-10, MCP-4, MDC, MIP-1α, MIP-1β and TARC are higher in the plasma of CL patients as compared to controls. This is in agreement with the study by de Mesquita et al. that showed that in patients with *L. guyanensis* infection, Eotaxin, IL-8, IP-10, MCP-1, MIP-1α, MIP-1β were increased as compared to controls; and that of Vargas-Inchaustegui et al., that showed that IP-10 and MIP-1β were also increased in patients infected with *L. braziliensis* [56]. Our results also show that there were no differences in plasma chemokine levels between C LCL, S LCL and MCL patients. However, as compared to HNEC, S LCL patients had similar levels of MCP-4 and S LCL and MCL patients had similar levels of MIP-1α; suggesting that there are differences in some chemokine levels between HNEC and CL patients with different clinical presentations. The increased chemokine levels we show in this study could be indicative of both wound healing or uncontrolled inflammation [57–59].

We also show that there were no differences in IFN-γ and TNF-α levels in the plasma of CL patients and controls. This is in contradiction to different studies, e.g., Castellano et al. [60], Espir et al. [61] and de Mesquita et al. [26] that showed high levels of these cytokines during active CL. These differences might be due to differences in infecting parasites, in the number of patients tested and importantly, to differences in clinical characteristics of the CL patients. In most cases, CL patients from studies such as the references of [26, 56, 60, 61] present with a variety of different ages. Often, the CL cohorts are not characterised in detail, with no information about their BMI, duration of illness or number of lesions. The classification of the type of lesions is also complex. Our results show a lot of heterogeneity in patients age, the duration of their illness, the number of lesions and their BMI. These are all factors that might influence the immune response. For example, TNF-α [62], IFN-γ [63], IP-10 [64] and MCP-1 [65] have been shown to increase with age. And indeed, we found positive correlation between age and MCP-1 and IP-10 in C LCL patients. The levels of cytokines and chemokines are also likely to be influenced by the duration of illness: a study by Melby et al. showed increased levels of IL-1α, TNF-α, IL-10, and TGF-β mRNAs in older lesions [66]. The nutritional status of the host also impacts on the levels of cytokines: we have previously shown that malnutrition results in altered cytokine profiles, with negative correlations between BMI and cytokines such as TNF-α and IFN-γ [67].

We also found negative correlations between the parasite gradings and Eotaxin, Eotaxin-3 and MCP-1 in MCL patients. MCL lesions are characterised by low parasite numbers [68] and indeed, the grading in the large majority of the lesions in our study were 1 + and 2 +, with only one patient with a 3 +  and one patient with a 4 +. It also should be noted that the parasite grading might not be an accurate representation of the number of parasites in the lesions, as it is not been established whether parasites are distributed equally at the border of the lesions, where the scraping are collected.

The main limitation of this study was the low number of parasites isolated from MCL and DCL, as compared to LCL patients. We were also not able to assess the plasma cytokine and chemokine profile for DCL patients and compare those to the other clinical presentations (S and C LCL and MCL), as we did not collect plasma from DCL patients. Furthermore, due to the political unrest, it was not possible to follow-up the patients and we could therefore not conclude whether some of the lesions, especially those from C LCL and S LCL patients, were healing or nonhealing lesions.

## Conclusions

Our study did not identify parasite genetic factors or a systemic immune signature associated with different clinical presentation of CL. This study is the first to investigate the systemic immune response in patients presenting with different clinical presentations of CL caused by *L. aethiopica* in Gayint. We did not identify any differences in plasma chemokines and cytokines among the different forms of CL. This lack of difference might be explained by the fact that we measured a systemic response in the plasma. Measuring the immune response at the site of pathology, within the lesions, is likely to be more informative. However, we did identify a distinct systemic chemokine signature in CL patients compared to HNEC. These results show that changes in the systemic immune response are clearly detectable in the plasma of CL patients. Testing a broader array of chemokines and cytokines in both the plasma and the lesions might help identify an immune signature specific to the different clinical presentations of CL.

Identifying parasite genetic factors or immunological signatures associated with the various clinical presentations of CL will require the recruitment of large cohorts–particularly for the rarer presentations. Recruiting such cohorts in Ethiopia will be challenging, as CL primarily affects rural communities in highland areas that are difficult to access [69]. By establishing either immunological differences between healing and persistent lesions or genetic markers for parasites likely to cause persistent lesions, it might be possible to identify patients at risk of developing severe, persistent CL lesions early and ensure that they receive prompt treatment.

## Supplementary Information

Additional file 1: Figure S1. Genome-wide association between LCL and MCL presentation phenotypes and SNP variants in Gayint *L. aethiopica*. (A) Manhattan plot of p-values for association between LCL vs MCL presentation and SNP variants. Each point represents a single SNP variation, with position on x-axis indicating position on genome. Colours distinguish SNPs on different chromosomes. (B) QQ-plot of -ve log (base 10) of expected p-value under null hypothesis vs observed p-values. Each point represents association between a SNP variant and LCL vs MCL presentation phenotype as shown in panel A, ordered by significance.

Additional file 2.

Additional file 3.

## Data Availability

Genomic data generated during the current study are available from the European Nucleotide Archive repository, with accession numbers for each sample listed in Table S2.

## References

[1] Burza S, Croft SL, Boelaert M. Leishmaniasis. Lancet. 2018;392(10151):951–70. 10.1016/S0140-6736(18)31204-2 30126638

[2] Steverding D. The history of leishmaniasis. Parasit Vectors. 2017;10(1):82. 28202044 10.1186/s13071-017-2028-5 PMC5312593

[3] van Henten S, Adriaensen W, Fikre H, Akuffo H, Diro E, Hailu A, et al. Cutaneous leishmaniasis due to *Leishmania aethiopica*. EClinicalMedicine. 2018;6:69–81. 31193672 10.1016/j.eclinm.2018.12.009 PMC6537575

[4] Ashford RW, Bray MA, Hutchinson MP, Bray RS. The epidemiology of cutaneous leishmaniasis in Ethiopia. Trans R Soc Trop Med Hyg. 1973;67(4):568–601. 4150462 10.1016/0035-9203(73)90088-6

[5] Lemma W, Erenso G, Gadisa E, Balkew M, Gebre-Michael T, Hailu A. A zoonotic focus of cutaneous leishmaniasis in Addis Ababa, Ethiopia. Parasit Vectors. 2009;2(1):60. 19954530 10.1186/1756-3305-2-60 PMC2794267

[6] Guidelines for diagnosis, treatment and prevention of leishmaniasis in Ethiopia, (2013). https://www.afrikadia.org/wp-content/uploads/2018/08/VL_Guidelines_Ethiopia_2013.pdf. Accessed 15 August 2024

[7] Lypaczewski P, Zhang WW, Matlashewski G. Evidence that a naturally occurring single nucleotide polymorphism in the RagC gene of *Leishmania donovani* contributes to reduced virulence. PLoS Negl Trop Dis. 2021;15(2):e0009079. 33621241 10.1371/journal.pntd.0009079 PMC7901767

[8] Cysne-Finkelstein L, Silva-Almeida M, Pereira BAS, Dos Santos CK, Bertho AL, Bastos LS, et al. Evidence of subpopulations with distinct biological features within a *Leishmania (Viannia) braziliensis* strain. Protist. 2018;169(1):107–21. 29482071 10.1016/j.protis.2017.11.004

[9] Banuls AL, Hide M, Prugnolle F. *Leishmania* and the leishmaniases: a parasite genetic update and advances in taxonomy, epidemiology and pathogenicity in humans. Adv Parasitol. 2007;64:1–109. 17499100 10.1016/S0065-308X(06)64001-3

[10] Acestor N, Masina S, Ives A, Walker J, Saravia NG, Fasel N. Resistance to oxidative stress is associated with metastasis in mucocutaneous leishmaniasis. J Infect Dis. 2006;194(8):1160–7. 16991092 10.1086/507646

[11] Cuervo P, Cupolillo E, Nehme N, Hernandez V, Saravia N, Fernandes O. *Leishmania (Viannia)*: genetic analysis of cutaneous and mucosal strains isolated from the same patient. Exp Parasitol. 2004;108(1–2):59–66. 15491550 10.1016/j.exppara.2004.07.005

[12] Jaber HT, Hailu A, Pratlong F, Lami P, Bastien P, Jaffe CL. Analysis of genetic polymorphisms and tropism in East African *Leishmania donovani* by amplified fragment length polymorphism and kDNA minicircle sequencing. Infect Genet Evol. 2018;65:80–90. 30016714 10.1016/j.meegid.2018.07.016 PMC6218636

[13] Downing T, Imamura H, Decuypere S, Clark TG, Coombs GH, Cotton JA, et al. Whole genome sequencing of multiple *Leishmania donovani* clinical isolates provides insights into population structure and mechanisms of drug resistance. Genome Res. 2011;21(12):2143–56. 22038251 10.1101/gr.123430.111 PMC3227103

[14] Van den Broeck F, Savill NJ, Imamura H, Sanders M, Maes I, Cooper S, et al. Ecological divergence and hybridization of Neotropical *Leishmania* parasites. Proc Natl Acad Sci USA. 2020;117(40):25159–68. 32958676 10.1073/pnas.1920136117 PMC7547230

[15] Franssen SU, Durrant C, Stark O, Moser B, Downing T, Imamura H, et al. Global genome diversity of the *Leishmania donovani* complex. Elife. 2020;9:e51243. 32209228 10.7554/eLife.51243 PMC7105377

[16] Grace CA, Sousa Carvalho KS, Sousa Lima MI, Costa Silva V, Reis-Cunha JL, Brune MJ, et al. Parasite genotype is a major predictor of mortality from visceral leishmaniasis. MBio. 2022;13(6):e0206822. 36222512 10.1128/mbio.02068-22 PMC9765272

[17] Hadermann A, Heeren S, Maes I, Dujardin JC, Domagalska MA, Van den Broeck F. Genome diversity of *Leishmania aethiopica*. Front Cell Infect Microbiol. 2023;13:1147998. 37153154 10.3389/fcimb.2023.1147998 PMC10157169

[18] Farias Amorim C, Lovins VM, Singh TP, Novais FO, Harris JC, Lago AS, et al. Multiomic profiling of cutaneous leishmaniasis infections reveals microbiota-driven mechanisms underlying disease severity. Sci Transl Med. 2023;15(718):eadh1469. 37851822 10.1126/scitranslmed.adh1469 PMC10627035

[19] Stamper LW, Patrick RL, Fay MP, Lawyer PG, Elnaiem DE, Secundino N, et al. Infection parameters in the sand fly vector that predict transmission of *Leishmania major*. PLoS Negl Trop Dis. 2011;5(8):e1288. 21886852 10.1371/journal.pntd.0001288 PMC3160291

[20] Castellucci LC, Almeida LF, Jamieson SE, Fakiola M, Carvalho EM, Blackwell JM. Host genetic factors in American cutaneous leishmaniasis: a critical appraisal of studies conducted in an endemic area of Brazil. Mem Inst Oswaldo Cruz. 2014;109(3):279–88. 24863979 10.1590/0074-0276140028 PMC4131779

[21] Novais FO, Amorim CF, Scott P. Host-directed therapies for cutaneous leishmaniasis. Front Immunol. 2021;12:660183. 33841444 10.3389/fimmu.2021.660183 PMC8032888

[22] Sacks DL, Noben-Trauth N. The immunology of susceptibility and resistance to *Leishmania major* in mice. Nature Reviews Immunol. 2002;2:845–58. 10.1038/nri933 12415308

[23] Kaye P, Scott P. Leishmaniasis: complexity at the host-pathogen interface. Nat Rev Microbiol. 2011;9(8):604–15. 21747391 10.1038/nrmicro2608

[24] Rossi M, Fasel N. The criminal association of *Leishmania* parasites and viruses. Curr Opin Microbiol. 2018;46:65–72. 30096485 10.1016/j.mib.2018.07.005

[25] Coutinho SG, Da-Cruz AM, Bertho AL, Santiago MA, De-Luca P. Immunologic patterns associated with cure in human American cutaneous leishmaniasis. Braz J Med Biol Res. 1998;31(1):139–42. 9686191 10.1590/s0100-879x1998000100019

[26] de Mesquita TGR, Junior J, da Silva LDO, Silva GAV, de Araujo FJ, Pinheiro SK, et al. Distinct plasma chemokines and cytokines signatures in *Leishmania guyanensis*-infected patients with cutaneous leishmaniasis. Front Immunol. 2022;13:974051. 36091007 10.3389/fimmu.2022.974051 PMC9453042

[27] Da-Cruz AM, Bittar R, Mattos M, Oliveira-Neto MP, Nogueira R, Pinho-Ribeiro V, et al. T-cell-mediated immune responses in patients with cutaneous or mucosal leishmaniasis: long-term evaluation after therapy. Clin Diagn Lab Immunol. 2002;9(2):251–6. 11874860 10.1128/CDLI.9.2.251-256.2002 PMC119941

[28] Bacellar O, Lessa H, Schriefer A, Machado P, Ribeiro de Jesus A, Dutra WO, et al. Up-regulation of Th1-type responses in mucosal leishmaniasis patients. Infect Immun. 2002; 70(12):6734–40. 10.1128/IAI.70.12.6734-6740.2002 PMC132996 12438348

[29] Convit J, Pinardi ME, Rondon AJ. Diffuse cutaneous leishmaniasis: a disease due to an immunological defect of the host. Trans R Soc Trop Med Hyg. 1972;66(4):603–10. 5071089 10.1016/0035-9203(72)90306-9

[30] Akuffo H, Schurr E, Andersson G, Yamaneberhan T, Britton S. Responsiveness in diffuse versus local cutaneous leishmaniasis is due to parasite differences. Scand J Immunol. 1987;26(6):717–21. 3122314 10.1111/j.1365-3083.1987.tb02308.x

[31] Akuffo H, Maasho K, Blostedt M, Hojeberg B, Britton S, Bakhiet M. *Leishmania aethiopica* derived from diffuse leishmaniasis patients preferentially induce mRNA for interleukin-10 while those from localized leishmaniasis patients induce interferon-gamma. J Infect Dis. 1997;175(3):737–41. 9041358 10.1093/infdis/175.3.737

[32] Chanyalew M, Abebe M, Endale B, Girma S, Tasew G, Bobosha K, et al. Enhanced activation of blood neutrophils and monocytes in patients with Ethiopian localized cutaneous leishmaniasis in response to *Leishmania aethiopica* neutrophil activation in Ethiopian cutaneous leishmaniasis. Acta Trop. 2021;220:105967. 34029532 10.1016/j.actatropica.2021.105967

[33] Pisa P, Gennene M, Soder O, Ottenhoff T, Hansson M, Kiessling R. Serum tumor necrosis factor levels and disease dissemination in leprosy and leishmaniasis. J Infect Dis. 1990;161(5):988–91. 2324549 10.1093/infdis/161.5.988

[34] Yizengaw E, Gashaw B, Yimer M, Takele Y, Nibret E, Yismaw G, et al. Demographic characteristics and clinical features of patients presenting with different forms of cutaneous leishmaniasis, in Lay Gayint, Northern Ethiopia. medRxiv. 2024. 10.1101/2024.02.15.24302809. Accessed 15 August 2024. 10.1371/journal.pntd.0012409 PMC11349221 39146362

[35] Kozarewa I, Ning Z, Quail MA, Sanders MJ, Berriman M, Turner DJ. Amplification-free Illumina sequencing-library preparation facilitates improved mapping and assembly of (G+C)-biased genomes. Nat Methods. 2009;6(4):291–5. 19287394 10.1038/nmeth.1311 PMC2664327

[36] Bolger AM, Lohse M, Usadel B. Trimmomatic: a flexible trimmer for Illumina sequence data. Bioinformatics. 2014;30(15):2114–20. 24695404 10.1093/bioinformatics/btu170 PMC4103590

[37] Warren WC, Akopyants NS, Dobson DE, Hertz-Fowler C, Lye LF, Myler PJ, et al. Genome Assemblies across the diverse evolutionary spectrum of *Leishmania* protozoan parasites. Microbiol Resour Announc. 2021;10(35):e0054521. 34472979 10.1128/MRA.00545-21 PMC8411921

[38] Shanmugasundram A, Starns D, Bohme U, Amos B, Wilkinson PA, Harb OS, et al. TriTrypDB: an integrated functional genomics resource for kinetoplastida. PLoS Negl Trop Dis. 2023;17(1):e0011058. 36656904 10.1371/journal.pntd.0011058 PMC9888696

[39] Li H, Durbin R. Fast and accurate short read alignment with Burrows-Wheeler transform. Bioinformatics. 2009;25(14):1754–60. 19451168 10.1093/bioinformatics/btp324 PMC2705234

[40] Danecek P, Bonfield JK, Liddle J, Marshall J, Ohan V, Pollard MO, et al. Twelve years of SAMtools and BCFtools. Gigascience. 2021;10(2):giab008. 33590861 10.1093/gigascience/giab008 PMC7931819

[41] Kozlov AM, Darriba D, Flouri T, Morel B, Stamatakis A. RAxML-NG: a fast, scalable and user-friendly tool for maximum likelihood phylogenetic inference. Bioinformatics. 2019;35(21):4453–5. 31070718 10.1093/bioinformatics/btz305 PMC6821337

[42] Yu G. Using ggtree to visualize data on tree-like structures. Curr Protoc Bioinformatics. 2020;69(1):e96. 32162851 10.1002/cpbi.96

[43] Purcell S, Neale B, Todd-Brown K, Thomas L, Ferreira MA, Bender D, et al. PLINK: a tool set for whole-genome association and population-based linkage analyses. Am J Hum Genet. 2007;81(3):559–75. 17701901 10.1086/519795 PMC1950838

[44] Uffelmann E, Huang QQ, Munung NS, de Vries J, Okada Y, Martin AR, et al. Genome-wide association studies. Nature Rev Methods Primers. 2021;1(1):59.

[45] Wickham H, Averick M, Bryan J, Chang W, McGowan LD, François R, et al. Welcome to the tidyverse. J Open Sour Softw. 2019;4(43):1686.

[46] Chulay JD, Bryceson AD. Quantitation of amastigotes of *Leishmania donovani* in smears of splenic aspirates from patients with visceral leishmaniasis. Am J Trop Med Hyg. 1983;32(3):475–9. 6859397 10.4269/ajtmh.1983.32.475

[47] Franssen SU, Takele Y, Adem E, Sanders MJ, Muller I, Kropf P, et al. Diversity and within-host evolution of *Leishmania donovani* from visceral leishmaniasis patients with and without HIV coinfection in Northern Ethiopia. MBio. 2021;12(3):e0097121. 34182785 10.1128/mBio.00971-21 PMC8262925

[48] Imamura H, Downing T, Van den Broeck F, Sanders MJ, Rijal S, Sundar S, et al. Evolutionary genomics of epidemic visceral leishmaniasis in the Indian subcontinent. Elife. 2016;5:e12613. 27003289 10.7554/eLife.12613 PMC4811772

[49] Krayter L, Schnur LF, Schonian G. The genetic relationship between *Leishmania aethiopica* and *Leishmania tropica* revealed by comparing microsatellite profiles. PLoS ONE. 2015;10(7):e0131227. 26196393 10.1371/journal.pone.0131227 PMC4511230

[50] Schönian G, Akuffo H, Lewin S, Maasho K, Nylen S, Pratlong F, et al. Genetic variability within the species *Leishmania aethiopica* does not correlate with clinical variations of cutaneous leishmaniasis. Mol Biochem Parasitol. 2000;239:239–48. 10.1016/s0166-6851(99)00216-9 10699253

[51] Amare GA, Mekonnen GG, Kassa M, Addisu A, Kendie DA, Tegegne B, et al. First report of cutaneous leishmaniasis caused by *Leishmania donovani* in Ethiopia. Parasit Vectors. 2023;16(1):457. 38104111 10.1186/s13071-023-06057-9 PMC10725588

[52] Elamin EM, Guizani I, Guerbouj S, Gramiccia M, El Hassan AM, Di Muccio T, et al. Identification of *Leishmania donovani* as a cause of cutaneous leishmaniasis in Sudan. Trans R Soc Trop Med Hyg. 2008;102(1):54–7. 18037149 10.1016/j.trstmh.2007.10.005

[53] Mebrahtu YB, Van Eys G, Guizani I, Lawyer PG, Pamba H, Koech D, et al. Human cutaneous leishmaniasis caused by *Leishmania donovani* s.l. in Kenya. Trans R Soc Trop Med Hyg. 1993;87(5):598–601. 8266420 10.1016/0035-9203(93)90101-u

[54] Pratlong F, Bastien P, Perello R, Lami P, Dedet JP. Human cutaneous leishmaniasis caused by *Leishmania donovani* sensu stricto in Yemen. Trans R Soc Trop Med Hyg. 1995;89(4):398–9. 7570877 10.1016/0035-9203(95)90025-x

[55] Aly R, Argaman M, Halman S, Shapira M. A regulatory role for the 5’ and 3’ untranslated regions in differential expression of hsp83 in *Leishmania*. Nucleic Acids Res. 1994;22(15):2922–9. 8065903 10.1093/nar/22.15.2922 PMC310256

[56] Vargas-Inchaustegui DA, Hogg AE, Tulliano G, Llanos-Cuentas A, Arevalo J, Endsley JJ, et al. CXCL10 production by human monocytes in response to *Leishmania braziliensis* infection. Infect Immun. 2010;78(1):301–8. 19901067 10.1128/IAI.00959-09 PMC2798186

[57] Rees PA, Greaves NS, Baguneid M, Bayat A. Chemokines in wound healing and as potential therapeutic targets for reducing cutaneous scarring. Adv Wound Care (New Rochelle). 2015;4(11):687–703. 26543682 10.1089/wound.2014.0568 PMC4620529

[58] Ridiandries A, Tan JTM, Bursill CA. The role of chemokines in wound healing. Int J Mol Sci. 2018;19(10):3217. 30340330 10.3390/ijms19103217 PMC6214117

[59] Le Y, Zhou Y, Iribarren P, Wang J. Chemokines and chemokine receptors: their manifold roles in homeostasis and disease. Cell Mol Immunol. 2004;1(2):95–104. 16212895

[60] Castellano LR, Filho DC, Argiro L, Dessein H, Prata A, Dessein A, et al. Th1/Th2 immune responses are associated with active cutaneous leishmaniasis and clinical cure is associated with strong interferon-gamma production. Hum Immunol. 2009;70(6):383–90. 19480861 10.1016/j.humimm.2009.01.007

[61] Espir TT, Figueira Lde P, Naiff Mde F, da Costa AG, Ramalho-Ortigao M, Malheiro A, et al. The role of inflammatory, anti-inflammatory, and regulatory cytokines in patients infected with cutaneous leishmaniasis in Amazonas State. Brazil J Immunol Res. 2014;2014:481750. 25295285 10.1155/2014/481750 PMC4177821

[62] Alberro A, Iribarren-Lopez A, Saenz-Cuesta M, Matheu A, Vergara I, Otaegui D. Inflammaging markers characteristic of advanced age show similar levels with frailty and dependency. Sci Rep. 2021;11(1):4358. 33623057 10.1038/s41598-021-83991-7 PMC7902838

[63] Bandres E, Merino J, Vazquez B, Inoges S, Moreno C, Subira ML, et al. The increase of IFN-gamma production through aging correlates with the expanded CD8(+high)CD28(-)CD57(+) subpopulation. Clin Immunol. 2000;96(3):230–5. 10964541 10.1006/clim.2000.4894

[64] Miles EA, Rees D, Banerjee T, Cazzola R, Lewis S, Wood R, et al. Age-related increases in circulating inflammatory markers in men are independent of BMI, blood pressure and blood lipid concentrations. Atherosclerosis. 2008;196(1):298–305. 17118371 10.1016/j.atherosclerosis.2006.11.002

[65] Inadera H, Egashira K, Takemoto M, Ouchi Y, Matsushima K. Increase in circulating levels of monocyte chemoattractant protein-1 with aging. J Interferon Cytokine Res. 1999;19(10):1179–82. 10547158 10.1089/107999099313127

[66] Melby PC, Andrade-Narvaez FJ, Darnell BJ, Valencia-Pacheco G, Tryon VV, Palomo-Cetina A. Increased expression of proinflammatory cytokines in chronic lesions of human cutaneous leishmaniasis. Infect Immun. 1994;62(3):837–42. 8112853 10.1128/iai.62.3.837-842.1994 PMC186190

[67] Takele Y, Adem E, Getahun M, Tajebe F, Kiflie A, Hailu A, et al. Malnutrition in healthy individuals results in increased mixed cytokine profiles, altered neutrophil subsets and function. PLoS ONE. 2016;11(8):e0157919. 27548305 10.1371/journal.pone.0157919 PMC4993519

[68] Jara M, Adaui V, Valencia BM, Martinez D, Alba M, Castrillon C, et al. Real-time PCR assay for detection and quantification of *Leishmania (Viannia)* organisms in skin and mucosal lesions: exploratory study of parasite load and clinical parameters. J Clin Microbiol. 2013;51(6):1826–33. 23554201 10.1128/JCM.00208-13 PMC3716068

[69] Lemma A, Foster WA, Gemetchu T, Preston PM, Bryceson A, Minter DM. Studies on leishmaniasis in Ethiopia. I. Preliminary investigations into the epidemiology of cutaneous leishmaniasis in the highlands. Ann Trop Med Parasitol. 1969;63(4):455–72. 5394018

