# Supplementary figures and images for: Investigation of parasite genetic variation and systemic immune responses in patients presenting with different clinical presentations of cutaneous leishmaniasis caused by Leishmania aethiopica
Source: Infect Dis Poverty. 2024 Oct 16;13:76. doi: 10.1186/s40249-024-01244-x (PMC11484111; doi:10.1186/s40249-024-01244-x)

A

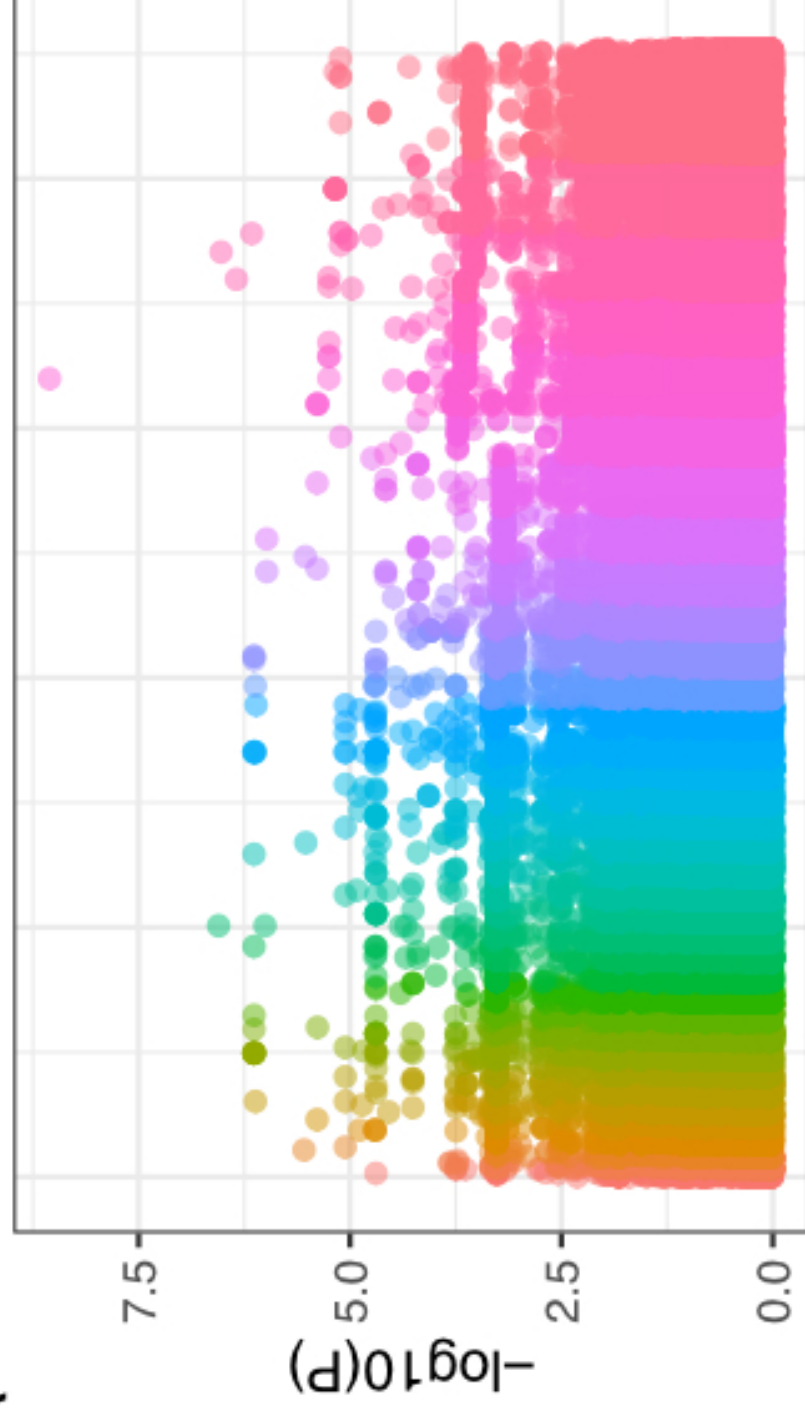

B

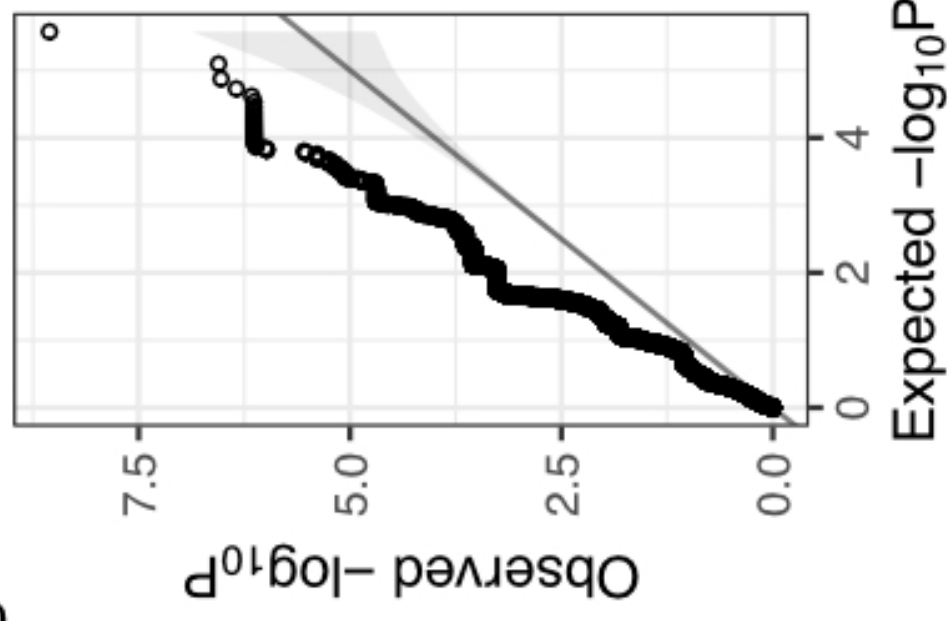

Figure S1

genomic position

Supplement: Supplementary file 1 — Additional file 1: Figure S1. Genome-wide association between LCL and MCL presentation phenotypes and SNP variants in Gayint L. aethiopica. (A) Manhattan plot of p-values for association between LCL vs MCL presentation and SNP variants. Each point represents a single SNP variation, with position on x-axis indicating position on genome. Colours distinguish SNPs on different chromosomes. (B) QQ-plot of -ve log (base 10) of expected p-value under null hypothesis vs observed p-values. Each point represents association between a SNP variant and LCL vs MCL presentation phenotype as shown in panel A, ordered by significance. [file 40249_2024_1244_MOESM1_ESM.pdf]

A

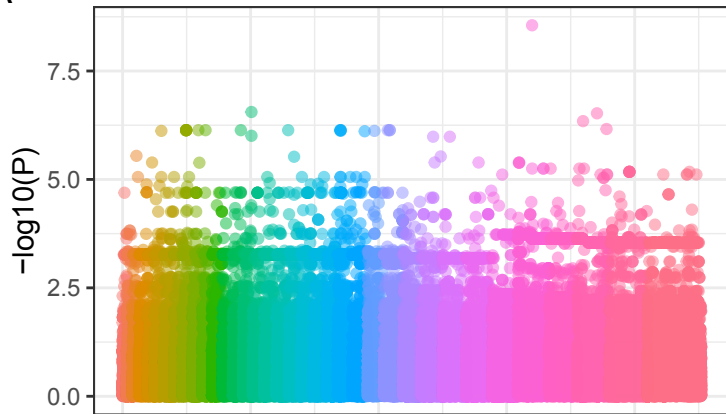

Figure S1

genomic position

B

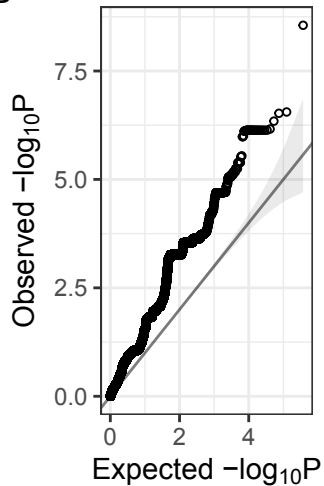

Supplement: Supplementary file 2 — Additional file 2. [file 40249_2024_1244_MOESM2_ESM.pdf]
