# Supplementary material for: Investigation of parasite genetic variation and systemic immune responses in patients presenting with different clinical presentations of cutaneous leishmaniasis caused by Leishmania aethiopica
Source: Infect Dis Poverty. 2024 Oct 16;13:76. doi: 10.1186/s40249-024-01244-x (PMC11484111; doi:10.1186/s40249-024-01244-x)
Supplement: Supplementary file 3 — Additional file 3. [file 40249_2024_1244_MOESM3_ESM.docx]

**Table S1: Test for normality of the datasets**

|  | CL | HNEC | C LCL | S LCL | MCL |
| --- | --- | --- | --- | --- | --- |
|  | *^P*-value | *^P*-value | *^P*-value | *^P*-value | *^P*-value |
| Eotaxin | < 0.0001 | 0.1426 | < 0.0001 | < 0.0001 | 0.0024 |
| Eotaxin 3 | < 0.0001 | 0.0014 | < 0.0001 | < 0.0001 | 0.0003 |
| IL-8 | < 0.0001 | 0.0300 | < 0.0001 | < 0.0001 | < 0.0001 |
| IP-10 | < 0.0001 | < 0.0001 | < 0.0001 | 0.0001 | < 0.0001 |
| MCP-1 | < 0.0001 | 0.5359 | < 0.0001 | < 0.0001 | 0.0254 |
| MCP-4 | < 0.0001 | 0.0020 | 0.0001 | < 0.0001 | 0.0033 |
| MDC | < 0.0001 | 0.0490 | < 0.0001 | 0.0022 | 0.0010 |
| MIP-1α | < 0.0001 | 0.9896 | < 0.0001 | < 0.0001 | 0.9236 |
| MIP-1β | < 0.0001 | 0.0079 | < 0.0001 | < 0.0001 | 0.0028 |
| TARC | < 0.0001 | 0.0014 | < 0.0001 | 0.0002 | < 0.0001 |
| IFN-γ | < 0.0001 | < 0.0001 | < 0.0001 | < 0.0001 | < 0.0001 |
| TNF-α | < 0.0001 | 0.4556 | < 0.0001 | < 0.0001 | < 0.0001 |
| Age | < 0.0001 | N/T | 0.0002 | 0.0011 | 0.0086 |
| BMI | 0.8141 | N/T | 0.6930 | 0.0952 | 0.0260 |
| Number of lesions | < 0.0001 | N/A | < 0.0001 | < 0.0001 | < 0.0001 |
| Parasite gradings | < 0.0001 | N/A | < 0.0001 | < 0.0001 | < 0.0001 |
| Duration of illness | < 0.0001 | N/A | < 0.0001 | < 0.0001 | < 0.0001 |

The Shapiro-Wilk test was used to assess the distribution of the data sets. Cytokine and chemokine levels were measured in pg/ml, age in years, BMI in kg/m^2^, parasite gradings in (+) and duration of illness in months. *CL* cutaneous leishmaniasis; *HNEC* healthy nonendemic controls; *C LCL* contained localised cutaneous leishmaniasis; *S LCL* spreading localised cutaneous leishmaniasis; *MCL* mucocutaneous leishmaniasis;

*N/T* not tested. *N/A* not applicable.

**Table S2 – Samples used in genetic analysis**

| Sample name | Phenotype | Number of pairs of reads generated | Total Sequence Data generated (bp) | ENA sample accession number |
| --- | --- | --- | --- | --- |
| 32 | LCL | 17516120 | 2644934120 | ERS6367640 |
| 33 | LCL | 15088118 | 2278305818 | ERS6367626 |
| 37 | LCL | 16390578 | 2474977278 | ERS6367642 |
| 38 | LCL | 17555242 | 2650841542 | ERS6367651 |
| 39 | LCL | 19556730 | 2953066230 | ERS6367649 |
| 41 | LCL | 15855062 | 2394114362 | ERS6367655 |
| 43 | LCL | 16809078 | 2538170778 | ERS6367641 |
| 47 | LCL | 14953826 | 2258027726 | ERS6367633 |
| 50 | multiple | 19614396 | 2961773796 | ERS6367643 |
| 52 | LCL | 14918932 | 2252758732 | ERS6367647 |
| 54 | LCL | 17937616 | 2708580016 | ERS6367632 |
| 55 | LCL | 17550922 | 2650189222 | ERS6367634 |
| 60 | LCL | 19896486 | 3004369386 | ERS6367639 |
| 62 | LCL | 18447182 | 2785524482 | ERS6367645 |
| 63 | LCL | 17841260 | 2694030260 | ERS6367638 |
| 65 | MCL | 17136990 | 2587685490 | ERS6367652 |
| 68 | multiple | 17960148 | 2711982348 | ERS6367648 |
| C-01 | MCL | 16710894 | 2523344994 | ERS6367631 |
| C-02 | LCL | 18167926 | 2743356826 | ERS6367658 |
| C-03 | LCL | 18733882 | 2828816182 | ERS6367636 |
| C-07 | LCL | 21415936 | 3233806336 | ERS6367653 |
| C-16 | LCL | 18708912 | 2825045712 | ERS6367657 |
| C-20 | LCL | 24597900 | 3714282900 | ERS6367629 |
| C-21 | MCL | 20091276 | 3033782676 | ERS6367650 |
| C-28 | LCL | 17845426 | 2694659326 | ERS6367627 |
| C-29 | LCL | 18579430 | 2805493930 | ERS6367635 |
| C-30 | LCL | 16151408 | 2438862608 | ERS6367628 |
| C-31 | LCL | 16362374 | 2470718474 | ERS6367644 |
| C-34 | LCL | 20844586 | 3147532486 | ERS6367646 |
| C-35 | LCL | 20128524 | 3039407124 | ERS6367630 |
| C-38 | LCL | 15115736 | 2282476136 | ERS6367637 |
| C-39 | LCL | 17201404 | 2597412004 | ERS6367625 |
| C-41 | LCL | 17521432 | 2645736232 | ERS6367656 |
| C-43 | LCL | 17283186 | 2609761086 | ERS6367654 |
| 232 | LCL | 48800472 | 7368871272 | ERS13501735 |
| 229L | DCL | 55626720 | 8399634720 | ERS13501736 |
| 218 | MCL | 46393864 | 7005473464 | ERS13501737 |
| 217 | LCL | 45617724 | 6888276324 | ERS13501738 |
| 192L | MCL | 52175738 | 7878536438 | ERS13501739 |
| C221 | recidivans | 45224654 | 6828922754 | ERS13501725 |
| 229F | DCL | 52126450 | 7871093950 | ERS13501726 |
| 230 | LCL | 50540918 | 7631678618 | ERS13501728 |
| 231 | multiple | 47430720 | 7162038720 | ERS13501729 |
| C208 | multiple | 47544092 | 7179157892 | ERS13501730 |
| 223 | LCL | 46383638 | 7003929338 | ERS13501733 |
| C223 | LCL | 44568610 | 6729860110 | ERS13501731 |
| 222 | MCL | 38334880 | 5788566880 | ERS13501732 |
| 219 | LCL | 45523200 | 6874003200 | ERS13501734 |

**Table S3 – Association between chromosome somy variation and LCL and MCL presentation phenotypes.**

|  | Chr 1 | Chr 4 | Chr 6 | Chr 10 | Chr 15 | Chr 31 |
| --- | --- | --- | --- | --- | --- | --- |
| LCL vs MCL | 0.157 | 0.146 | 1 | 1 | 0.146 | 1 |
| S LCL vs C LCL | 0.724 | N/A | 0.353 | 0.602 | N/A | 0.687 |
| C LCL vs MCL | 0.182 | 0.214 | N/A | 1 | 0.214 | 1 |
| S LCL vs MCL | 0.421 | 0.333 | 1 | 0.529 | 0.333 | 0.615 |

Entries are *P*-values from Fisher’s exact test of association between estimated somy (chromosome dosage) and phenotype for all comparisons over which the somy varies within the set of samples with relevant phenotypes. N/A indicates a phenotype: chromosome combination for which all samples with those phenotypes have identical somy. For chromosomes not shown on the table, all samples have identical somy for all phenotype comparisons.

**Table S4: Plasma levels of TNF**α **in LCL and MCL patients**

|  | **LCL** | **MCL** | ***P*-value** |
| --- | --- | --- | --- |
| **TNF**α (pg/ml) | 0.84 (0.68–1.14) | 0.72 (0.57–0.85) | 0.0359 |

TNFα levels were measured in the plasma isolated from the blood of LCL (*n* = 96) and MCL (*n* = 33) by multiplex assay, as described in Materials and Methods.

The statistical difference between LCL and MCL was tested by Mann-Whitney. *LCL* localised cutaneous leishmaniasis; *MCL* mucocutaneous leishmaniasis.

**Table S5: Correlations between plasma chemokines and cytokines and the different clinical parameters**

|  | | C LCL | S LCL | MCL | Number of XY pairs (C/S/MCL) |
| --- | --- | --- | --- | --- | --- |
|  |  | **P*-value | **P*-value | **P*-value |  |
| Eotaxin  (pg/ml) | Age (years) | 0.1096 | 0.6916 | 0.7714 | 63/27/33 |
|  | BMI (kg/m^2^) | 0.7029 | 0.8030 | 0.3475 | 64/27/33 |
|  | Number of lesions | **0.0456** | 0.3037 | 0.6100 | 64/27/33 |
|  | Parasite grading (+) | 0.1122 | 0.3289 | **0.0120** | 46/23/24 |
|  | Duration of illness (months) | 0.9335 | 0.5055 | 0.7937 | 63/27/33 |
|  | | C LCL | S LCL | MCL |  |
|  |  | **P*-value | **P*-value | **P*-value |  |
| Eotaxin 3  (pg/ml) | Age (years) | 0.5737 | 0.9443 | 0.4677 | 63/27/33 |
|  | BMI (kg/m^2^) | 0.7731 | 0.6921 | 0.6404 | 64/27/33 |
|  | Number of lesions | 0.2601 | 0.5565 | 0.8251 | 64/27/33 |
|  | Parasite grading (+) | 0.1521 | 0.8682 | **0.0040** | 46/23/24 |
|  | Duration of illness (months) | 0.5133 | 0.8526 | 0.5631 | 63/27/33 |
|  | | C LCL | S LCL | MCL |  |
|  |  | **P*-value | **P*-value | **P*-value |  |
| IL-8  (pg/ml) | Age (years) | 0.9043 | 0.1546 | 0.9800 | 64/27/33 |
|  | BMI (kg/m^2^) | 0.2655 | 0.0509 | 0.8752 | 65/28/33 |
|  | Number of lesions | 0.7507 | 0.9530 | 0.7371 | 65/28/33 |
|  | Parasite grading (+) | 0.8956 | 0.7897 | 0.5931 | 46/23/24 |
|  | Duration of illness (months) | 0.5534 | 0.2648 | 0.1897 | 65/31/33 |
|  | | C LCL | S LCL | MCL |  |
|  |  | **P*-value | **P*-value | **P*-value |  |
| IP-10  (pg/ml) | Age (years) | **0.0089** | 0.8329 | 64/27/33 | 63/27/33 |
|  | BMI (kg/m^2^) | 0.4624 | 0.1868 | 64/27/33 | 64/27/33 |
|  | Number of lesions | 0.1032 | 0.5890 | 46/23/24 | 64/27/33 |
|  | Parasite grading (+) | 0.6989 | 0.9040 | 63/27/33 | 46/23/24 |
|  | Duration of illness (months) | 0.4956 | 0.7765 | 63/27/33 | 63/27/33 |
|  | | C LCL | S LCL | MCL |  |
|  |  | **P*-value | **P*-value | **P*-value |  |
| MCP-1  (pg/ml) | Age (years) | **0.0125** | 0.8590 | 0.6842 | 63/27/33 |
|  | BMI (kg/m^2^) | 0.1199 | 0.3310 | 0.8188 | 64/27/33 |
|  | Number of lesions | 0.0504 | 0.4132 | 0.4736 | 64/27/33 |
|  | Parasite grading (+) | 0.9772 | 0.2036 | **0.0112** | 46/23/24 |
|  | Duration of illness (months) | 0.8150 | 0.1811 | 0.4147 | 63/27/33 |
|  | | C LCL | S LCL | MCL |  |
|  |  | **P*-value | **P*-value | **P*-value |  |
| MCP4  (pg/ml) | Age (years) | 0.1624 | 0.6725 | 0.4205 | 63/27/33 |
|  | BMI (kg/m^2^) | 0.8876 | 0.8123 | 0.2277 | 64/27/33 |
|  | Number of lesions | 0.5351 | 0.4667 | 0.4536 | 64/27/33 |
|  | Parasite grading (+) | 0.1111 | 0.1630 | 0.1997 | 46/23/24 |
|  | Duration of illness (months) | 0.5535 | 0.4950 | 0.9342 | 63/27/33 |
|  | | C LCL | S LCL | MCL |  |
|  |  | **P*-value | **P*-value | **P*-value |  |
| MDC  (pg/ml) | Age (years) | 0.3940 | 0.2519 | 0.5324 | 63/27/33 |
|  | BMI (kg/m^2^) | 0.6868 | 0.8727 | 0.6605 | 64/27/33 |
|  | Number of lesions | 0.1355 | 0.9761 | 0.4150 | 64/27/33 |
|  | Parasite grading (+) | 0.7640 | 0.1961 | 0.8430 | 46/23/24 |
|  | Duration of illness (months) | 0.0584 | 0.7931 | 0.3951 | 63/27/33 |
|  | | C LCL | S LCL | MCL |  |
|  |  | **P*-value | **P*-value | **P*-value |  |
| MIP-1α  (pg/ml) | Age (years) | 0.9036 | 0.6353 | 0.6113 | 63/27/33 |
|  | BMI (kg/m^2^) | 0.7115 | 0.9216 | 0.2957 | 64/27/33 |
|  | Number of lesions | 0.4750 | 0.2288 | 0.1762 | 64/27/33 |
|  | Parasite grading (+) | 0.6886 | 0.5558 | 0.3333 | 46/23/24 |
|  | Duration of illness (months) | **0.0021** | 0.0685 | 0.1140 | 63/27/33 |
|  | | C LCL | S LCL | MCL |  |
|  |  | **P*-value | **P*-value | **P*-value |  |
| MIP-1β  (pg/ml) | Age | 0.3043 | 0.5911 | 0.7208 | 63/27/33 |
|  | BMI | 0.7017 | 0.3887 | 0.5920 | 64/27/33 |
|  | Number of lesions | 0.4757 | 0.3106 | 0.8785 | 64/27/33 |
|  | Parasite grading | 0.9775 | 0.5014 | 0.3120 | 46/23/24 |
|  | Duration of illness | 0.0963 | 0.0901 | 0.4747 | 63/27/33 |
|  | | C LCL | S LCL | MCL |  |
|  |  | **P*-value | **P*-value | **P*-value |  |
| TARC  (pg/ml) | Age (years) | 0.6603 | 0.4353 | 0.4727 | 63/27/33 |
|  | BMI (kg/m^2^) | 0.8581 | 0.3202 | 0.0850 | 64/27/33 |
|  | Number of lesions | 0.2912 | 0.8661 | 0.6915 | 64/27/33 |
|  | Parasite grading (+) | 0.8794 | 0.3526 | 0.4711 | 46/23/24 |
|  | Duration of illness (months) | 0.9889 | 0.6035 | 0.8109 | 63/27/33 |
|  | | C LCL | S LCL | MCL |  |
|  |  | **P*-value | **P*-value | **P*-value |  |
| IFNγ  (pg/ml) | Age (years) | 0.6087 | 0.0959 | 0.7957 | 64/27/33 |
|  | BMI (kg/m^2^) | 0.7280 | 0.9824 | 0.2122 | 65/28/33 |
|  | Number of lesions | 0.1438 | 0.6628 | 0.0871 | 65/28/33 |
|  | Parasite grading (+) | 0.9373 | 0.6538 | 0.4770 | 46/23/24 |
|  | Duration of illness (months) | 0.7366 | 0.0926 | 0.5314 | 65/31/33 |
|  | | C LCL | S LCL | MCL |  |
|  |  | **P*-value | **P*-value | **P*-value |  |
| TNFα  (pg/ml) | Age (years) | 0.6050 | 0.2273 | 0.8455 | 64/27/33 |
|  | BMI (kg/m^2^) | 0.8368 | 0.7639 | 0.5136 | 65/28/33 |
|  | Number of lesions | 0.1257 | 0.3701 | 0.9123 | 65/28/33 |
|  | Parasite grading (+) | 0.1721 | 0.2017 | 0.1489 | 46/23/24 |
|  | Duration of illness (months) | 0.2784 | 0.0752 | 0.4339 | 65/31/33 |

Correlations between the different clinical parameters and plasma chemokines and cytokines were measured by Spearman test. *MCL* mucocutaneous leishmaniasis; *C LCL* contained localised cutaneous leishmaniasis; *S LCL* spreading localised cutaneous leishmaniasis.
